# Schoolchildren’s motivation for viewing Chinese opera animation according to opera genre

**DOI:** 10.1371/journal.pone.0292744

**Published:** 2023-10-11

**Authors:** Chan Lv, Tzu-Fan Hsu, Xian-Feng Tu, Jia Li

**Affiliations:** 1 Ph.D. Program in Design, Chung Yuan Christian University, Taoyuan, Taiwan; 2 Department of Animation and Digital Media, School of Art, Wuyi University, Wuyishan, Fujian, China; 3 Institute of Creative Design and Management, National Taipei University of Business, Taipei, Taiwan; 4 Department of University Mathematics, School of Information Engineering, Jiaxing Nanhu University, Jiaxing, Zhejiang, China; 5 Department of Environment Design, School of Design, Jiaxing University, Jiaxing, Zhejiang, China; Novi Sad School of Business, SERBIA

## Abstract

The emergence of Chinese opera animation allows a wider audience, especially a younger audience, to access and embrace the art of opera heritage. This study used a two-way mixed-design ANOVA to explore the effect of Chinese opera animation on schoolchildren’s viewing motivation; the independent variables were the children’s grade level and the opera genre of the animation. Grade level was divided into three groups: lower, middle, and upper (grades 2, 4, and 6, respectively). Opera genre consisted of Peking, Yue, and Henan opera. The dependent variable, viewing motivation, comprised six dimensions: entertainment and relaxation, learning knowledge, escapist pastime, aesthetic appreciation, empathic identification, and socializing and sharing. After statistically analyzing the evaluations of 457 participants, the results showed the following: (1) Peking and Yue opera animation had a better entertainment and relaxation effect on the lower and middle groups. Henan opera had a better escapist pastime effect on the upper group but less effect on the lower group. (2) In terms of learning knowledge, empathic identification, aesthetic appreciation, and overall performance, Yue and Henan opera animations were more effective for enhancing viewing motivation compared with Peking opera animation. (3) The middle and lower groups showed higher viewing motivation than the upper group in the learning knowledge, empathic identification, and socializing and sharing dimensions. Overall, grades 2 and 4 were appropriate stages for schoolchildren to engage with opera animation. Our findings can provide a reference for promoting cultural heritage sustainability and support follow-up research on integrating opera animation into children’s education.

## Introduction and literature review

### Decline of Chinese opera culture

Chinese opera has a long history and is among the oldest theater cultures in the world [1, 2]. People have long admired Chinese opera for its colorful repertoire, high-quality performances, and unique ethnic style [1, 3]. There are more than 360 genres of opera in China [4]. Among them, Peking, Yue, and Henan opera are the top three genres in terms of the number of troupes, number of actors, and coverage area, as well as public and official recognition [4–6]. In 2010, Peking opera was added to UNESCO’s Representative List of the Intangible Cultural Heritage of Humanity; Henan opera and Yue opera have also been included in the national intangible cultural heritage list [4, 7]. Chinese opera is unique among the dramatic arts, known for its literary stories and use of poetry, the visual artistry of its masks and costumes, and its highly skilled dance and martial arts performances [8–10]. These characteristics make Chinese opera a cultural heritage worthy of preservation and development [8].

However, with the development of technology, the accelerated pace of life, and the diversification of media, the aesthetic interests of the public are constantly changing, and Chinese opera, which is mainly taught orally and performed on stage, is gradually losing its dominant position as a cultural tradition [11]. This crisis in Chinese opera began to manifest in the 1980s with the loss of audiences as well as the aging of audiences. Many theater companies have been disbanded, and certain types of opera are on the verge of extinction, giving rise to theories about the crisis and extinction of Chinese opera [12]. It has been suggested that to achieve modernization, Chinese opera needs to take into account the needs of contemporary audiences and keep up with the times; otherwise, Chinese opera will become a marginalized “refined art” and eventually be forgotten [13, 14]. Therefore, for Chinese opera to be sustainable, it is necessary to consider how to expand its audience, especially young audience, to increase its likelihood of being seen and recognized. With the development of digital media, various media arts and technologies have helped promote the dissemination of Chinese opera. In particular, opera animation is a new art form that has emerged in the process of modernizing Chinese opera, increasing its exposure to young audiences [15]. How to make full use of this new medium to attract young audiences, maintain its continuous development, and rejuvenate opera is an important consideration in the context of revitalizing Chinese opera and perpetuating cultural heritage.

### Chinese opera animation as cultural sustainability

One of the earliest forms of Chinese opera was shadow puppetry, which emerged about 2,000 years ago during the Western Han dynasty. Based on the performance principle of curtain and shadow, shadow puppetry performances made use of light and shadow effects, which would play a role in the early development of film and the emergence of animation [16, 17]. In 1941, the first Chinese animated feature film, *Princess Iron Fan*, used elements of opera in its character shapes and fight sequences. The 1948 puppet animation *Chang Hen Ge* incorporated Kunqu opera into its puppet animation. Later, in 1964, *Uproar in Heaven* showed an even deeper excavation of opera elements. Here, the Monkey King character was modeled after the “monkey face” in opera, and the character’s action was rooted in opera action. Moreover, the film’s music drew on opera elements such as recitative, which has become representative of the Chinese animation school [17–19]. Thus, the historical development of Chinese animation reveals that early Chinese animation deliberately absorbed and reproduced opera [1, 20], and this combination can also be considered the germination of contemporary Chinese opera animation (COA) [19].

In the twenty-first century, a new form of COA has emerged in response to the need to preserve, inherit, and develop traditional opera and broaden its audience to younger people. COA takes animation as the carrier, opera as the core element, and the opera repertoire as the basis for scripts. COA, moreover, retains the costumes, makeup, accents, genre characteristics, music, and performance style of opera. It aims to attract young audiences through animation so they can inherit the cultural tradition of opera [19, 21, 22]. To further develop COA and cultivate related creative talent, the Chinese Academy of Opera opened a new media art department in 2006 and established a professional art team. This group created significant COA works such as *Escape From The Temple* and *The Fifteen Strings of Coins*. Meanwhile, other institutions have also started to create COA. These include the 3D Digital Peking Opera Animation project between the Beijing Practical Senior Technical School and Bournemouth University, the Cartoon Chao Opera Troupe of Hanshan Normal University, Hubei University’s animation of the Chu Opera *Ge Ma*, and *the Broken Bridge* opera animation project of Zhejiang Sci-Tech University [19]. In 2005, the Chinese National Academy of Arts collaborated with Jiu Tian Xing Culture Communication Co. and Hunan Golden Eagle Culture Communication Co. to develop the Chinese Opera Classics Original Animation project. This project included 32 operas listed on the Intangible Cultural Heritage Protection List, including representatives of Peking, Kunqu, Yue, and Henan opera [18, 23, 24]. The first 100 episodes of the project, broadcast on China Central Television and China Education Television, targeted young audiences and focused on the fun of COA [21]. Such cooperation between government, academia, and business in the development of COA reflects the extent to which Chinese opera is being recognized and extended into the animation field in the current century [25].

Current research on COA mostly focuses on its creation methods and how to appropriately present opera via animation. There is a lack of research, however, on the audience for COA. COA is a type of popular art that aims to encourage more nontraditional fans, especially children, to pay attention to opera and thus accept and identify with Chinese opera art and culture [25–27]. It offers a way for them to acquire basic knowledge about Chinese opera at an early age, including the types and characteristics of opera genres, the meanings of facial makeup in Chinese opera, the classification of opera roles, and the function of opera props [28, 29]. Research on audiences is needed to understand the preferences and needs of young audiences and support the dissemination of COA. To support cultural preservation, and that of Chinese opera culture in particular, this study investigated COA with a focus on the main audience for animation: school-aged children [30]. By introducing COA into the learning process and observing schoolchildren’s motivational responses, we aim to provide a reference for COA creation and education while also increasing the possibility of broadening its audience.

### Appreciation age of child audiences

In communication science, an “audience” refers to “the unnamed individuals and groups to whom mass communication speaks” [31]. According to Erikson et al., children can be divided by age into infancy and early childhood before age six, childhood between seven and 12 years old, and late childhood above age 13, which is the period of transition to adolescence [32–34]. Each stage has its own characteristics, from simple to complex and from concrete to abstract. Since COA has specific artistic characteristics, such as singing, performance style, and suppositionality [35], it might not be understood and accepted by children of all ages [24, 36]. Therefore, understanding the stages of children’s psychological and cognitive development can help us identify the appropriate age for children to watch COA.

Lin suggested that children do not begin to understand and think about literary creation and the meanings of stories until after age four or five [37]. Jui and Sudrajat [36, 38] also noted that in language learning and development, children’s vocabulary increases rapidly after age five, when they become better able to think with abstract symbols. Kuo and Ningrum suggested that children aged seven to 11 can classify words in speaking contexts and understand and solve problems in the form of specific stories [39, 40]. Lv Hongmei and Yao Hailin noted that after entering school, children aged 7–12 are exposed to a richer social life and quickly form altruistic behaviors (sharing, cooperation, pro-sociality), resulting in a willingness to share and empathy [41].

According to Piaget [42], children have four cognitive stages: sensorimotor (0–2 years old), preoperational (2–7 years old), concrete operational (7–11 years old), and formal operational (11 years old and above). Thus, most elementary school children are in the concrete operational stage, in which they have an increasing desire for knowledge and can receive systematic knowledge. Wang et al. and Chen also note that school-age children have an improved ability to process information and that the amount of information they can retain increases steadily [43, 44]. Children at this stage have an attention span extending from 15 minutes at age five to 20–25 minutes at ages six and seven. The attention span of school-age children aged 7–12 can last up to 45 minutes for content with interesting images and situations [43, 44]. School-age children gradually learn how to think in abstract and logical ways compared with simply thinking in concrete images, thus promoting the initial formation of concepts and the ability to reason [40].

Primary school could be a good stage for cultivating children’s appreciation for opera. People who are exposed to opera during childhood are more likely to attend operas later in life [45–47]. Jui suggested that age 6–12 is the best time for children to learn to appreciate and analyze Chinese opera [36]. Studying children’s opera, Li Xu determined that simple and colorful opera works are more suitable for children at the middle and lower ages (5–10). This is because children at the higher age (10–16) tend to think in more logical ways while those at the middle and lower ages (5–10) tend to think more in images [48]. Generally, attention should be paid to introducing performing arts activities, such as classical music and opera, into education for children in the lower and middle grades [49]. Students in these grades are more open to new and varied art forms than those in higher grades [50, 51]. Students tend to show a gradually declining preference for such art forms as they get older [52].

Based on the above, we propose that COA is suitable for schoolchildren aged 6–12 who reach the average levels of physical and mental development. Children at this stage have more mature language skills and longer attention spans [39, 40, 43, 44]. This enables them to abstractly analyze and reason about the story content, suppositional performance style, and singing and recitation of COA, while also generating understanding, empathy, and learning [36]. Cheng and McClelland [53, 54], moreover, noted that at this developmental stage, children show considerable continuity in the development of habits, attitudes, interests, and personality traits, which can carry over into adolescent and adult development and have significant effects throughout the entire life-span.

### Thematic categories of Chinese opera genres

The development of Chinese opera was influenced by the cultural features of different places (e.g., dialects, music, and folklore), forming a unique aesthetic style that distinguishes it from other genres [55]. Since there are many genres of traditional Chinese opera with various themes, this study classifies the themes of COA based on the important representative genres of Chinese opera—namely, Peking opera, Yue opera, and Henan opera [4–6].

First, Peking opera is a comprehensive opera genre that includes both “gentle shows,” which are focused on singing and acting, and martial arts–focused shows and military plays, which are dominated by acrobatic fighting [56, 57]. There are more than 5,000 known plays in Peking opera [56]. Their content is taken from historical interpretations and folklore books, and they mostly include patriotic historical plays. Major themes include the Three Kingdoms theme, Yang Family General theme, Water Margin theme, Yue Family Army theme, Honest and Upright Official theme, the Mythology play, and The Dream of the Red Chamber theme [56, 58]. This is why Peking opera has been a long-standing reference for COA. For example, in The Proud General (1956), a pioneering work of the Chinese School of Animation, the shapes of the general and the master were borrowed from the face and action designs of the “Wu Sheng” (male martial arts role) and “Chou” (clown role) in Peking opera; it also used the gong and drums of opera music and the rhyming white elements of Peking opera [59]. The Peking opera themes of COA can be broadly divided into seven categories: patriotic theme, antifeudal oppression theme, honest and upright official theme, family ethics theme, love theme, gods and monsters theme, and folk life theme.

Second, since its inception, Yue opera has mostly included “gentle shows” (i.e., opera focused on singing and acting rather than martial arts) [57, 60]. Its repertoire inherits the traditions of Jiangnan culture [61]. Early Yue opera mainly included folk plays reflecting the lives of farmers. Later, the themes started to focused on love and family ethics [61, 62].

Finally, Henan opera is more oriented toward popular entertainment; it is generally easy to understand and rich in local characteristics. There are thousands of traditional plays in Henan opera, most of which are based on historical stories and novels. Themes include the Investiture of the Gods theme, Three Kingdoms theme, Wagang Army theme, Bao Gong theme, Yang Family General theme, and Yue Family Army theme. There are also some themes related to love, marriage, and morality [63]. For example, the first paper-cut animation, *Pigsy Eats Watermelon* (1958), incorporated the shadow play action style as well as the northern Henan ditty. The themes of Henan opera can be broadly divided as follows: patriotic, palace struggle, honest and upright official, antifeudal oppression, war, folk life, ethical and moral, mythical, and love [63, 64].

Some studies suggest that an audience’s preference for opera can be improved by having a better understanding of the opera genre and an increased familiarity with this art form [47, 65]. Children’s appreciation of different types of performing arts is influenced by participating in, listening to, and watching them [51, 66, 67]. It has been suggested that animation has positive effects on the learning processes of school-age children [68–70]. This is because animation can present complex topics in a simple form, which helps reduce cognitive burden [71]. Different genres of COA have their own characteristics, and their repertoire stories have different emphases. Therefore, we synthesized the aforementioned repertoires of Peking, Yue, and Henan opera and grouped the genre themes of COA into seven major categories: historical and patriotic, antifeudal oppression, honest and upright official, ethical and moral, love, gods and monsters, and folk life. Hence, we used these seven themes as the basis for the selection of COA stimuli to eliminate the influence of story theme on the measurement of viewing motivation.

### Motives for viewing COA

In psychology, motivation refers to an intrinsic drive that urges an individual toward explicit behaviors, causing the individual to continuously adopt goal-oriented behaviors. Motivation arises from stimuli and needs, which can drive an individual toward or away from a goal [72–74]. The stronger the motivation, the more the individual is inclined toward goal-oriented behavior [75, 76]. Motivation can vary from one individual to another due to different goals [77]. Viewing motivation is an inner driving force that promotes the individual’s viewing behavior [72]. “Viewing” means watching, participating in, or experiencing. The “viewer” refers to the audience or reader—that is, the people who participate in, watch, or experience a cultural activity, such as animation, opera, or sports [78]. Viewing motivation can drive the viewer’s initiative and active viewing behavior [79]. Viewing will become more frequent and a viewing habit will form when viewing motivation drives behavior and viewing motivation is satisfied [79]. Therefore, understanding an individual’s viewing motivation can help predict the direction and pattern of viewing behavior [80].

Uses and satisfactions theory considers the relationship between the viewer and the media from the audience’s perspective, mainly in terms of the viewer’s psychological and social needs, which produce the motivation to use media and lead to different media use behaviors, ultimately determining whether the viewer’s needs are satisfied [81–84]. Thus, an audience’s use of media is influenced by their needs and motivations, and whether the audience can be satisfied when using media determines their media expectations and their next media contact [85, 86]. COA, as a media product, hinges on whether its content can satisfy the interests and motivations of child viewers; thus, viewing motivation can be considered the driving force for child viewers to watch COA [86].

Viewing motives are multifaceted. He et al. divided children’s motivation for watching television into five categories: boredom, spending time with family, spending time with friends, learning useful knowledge, and improving academic performance. Ho and Hsu [88] proposed the animation viewing motivations of leisure and entertainment, relaxation, looking for excitement, knowledge and learning, appreciation of art, enrichment of the soul, dating and friendship, admiration of actors, looking for conversation topics, audience reviews, and other related aspects [88–91]. Lo and Cheng [92], meanwhile, categorized the motivations for watching animated news, a form of melodramatic animation, into relaxation, entertainment, information seeking, pass time, social interaction, interpersonal learning, and companionship [93, 94]. Regarding the motivation for viewing anime, Tseng identified six dimensions: knowledge, sense of identity, entertainment, friendship, catharsis, and content attraction [95–97]. In a study of child viewers, Jiang and Chen [86] determined six motivational dimensions of viewing animation: pastime companionship, learning and growth, habit dependence, pleasure and relaxation, aesthetic immersion, and social sharing [88, 98]. Lastly, Gonçalves et al. [99] categorized animation viewing communities in Portugal as follows: socio-informational, entertainment, passing time, escapist, and parasocial [100].

The facets of viewing motivation summarized in Table 1 can be further explained as follows:

1. Entertainment and relaxation: Studies have found that entertainment is an important motivation for viewers [83]. COA, as an animation medium, is entertaining in its own right. Thus, “relaxation and entertainment” is defined as one of the motives for children to view COA. This specifically includes leisure and relaxation, happiness, seeking excitement, and emotional enjoyment.
2. Learning knowledge: One the main reasons for creating COA is to transmit opera knowledge to the younger generation [25]. Therefore, from an educational standpoint, “learning knowledge” is included as the second motive; it includes getting information, learning new knowledge, satisfying curiosity, and gaining experience.
3. Escapist pastime: Haley noted that escapism plays an important role in movie and television viewing. Many of the abovementioned studies also identified escapism as a viewing motive. Thus, “escapist pastime” is the third motivation; it includes forgetting about your worries, passing the time, avoiding problems, and needing companionship [101].
4. Aesthetic appreciation: COA has specific artistic characteristics. Aesthetic perception can meet people’s need to seek knowledge, beauty, and pleasure [79]. Thus, we selected “aesthetic appreciation” as the fourth motivation. It includes story, picture style, characters, performance moves, and background music.
5. Empathic identification: The characters or plots of a movie can produce emotional resonance and self-identification in audiences. The audience’s empathy generates “illusionary intimacy” and thus creates enjoyment when watching a movie [102]. The closer the COA is to the audience’s personal thoughts, perceptions, and life experiences, the stronger this effect will be [95]. To understand whether COA brings traditional opera closer to current child audiences, “empathic identification” was defined as the fifth motivation. It includes character identification, value identity, emotional sustenance, emotional resonance, and self-inspiration.
6. Socializing and sharing: Media can broaden a viewer’s social needs and enhance interpersonal interaction through discussion with friends and family [79]. Therefore, we selected “socializing and sharing” as the sixth dimension. It includes establishing common interests, generating common topics, promoting emotional communication, and expanding friendships.

**Table 1:** Dimensions of viewing motivation.

	Entertainment and relaxation	Learning knowledge	Escapist pastime	Aesthetic appreciation	Empathic identification	Socializing and sharing
He et al. (2010) [87]		Learning useful knowledge, improving academic performance	Boredom			Spending time with family and friends
Ho & Hsu (2011) [88]	Leisure and entertainment, looking for excitement, relaxation	Knowledge and learning		Appreciation of art, admiration of actors	Enrichment of the soul	Dating and friendship, looking for conversation topics, audience reviews
Lo & Cheng (2013) [92]	Relaxation, entertainment	Information seeking	Pass time, companionship			Social interaction, interpersonal learning
Tseng (2017) [95]	Entertainment	Knowledge based	Catharsis	Content attraction	Sense of identity	Friendship
Jiang & Chen (2020) [86]	Pleasure and relaxation	Learning and growth	Pastime companionship, habit dependence	Aesthetics and aesthetic immersion		Social sharing
Gonçalves et al. (2021) [99]	Entertainment	Socio-informational	Pass time, escapist			Parasocial

### Research questions

COA has emerged in response to contemporary needs—namely, the current need to preserve and transmit traditional culture. There is a lack of research, however, on the audience for COA. Greenberg and Rubin found that there is a relationship between children’s age and their motivation to watch TV programs [103, 104]. Rubin suggests that there is a clear correlation between an audience’s motivation to watch TV shows and their content preferences [105]. This study, therefore, aimed to measure and analyze the viewing motivation of children in different age groups regarding opera animation based on Peking, Yue, and Henan opera. The findings can help expand the audience for COA and have significance for opera heritage and cultural perpetuation. The issues we aimed to explore can be summarized as follows:

We examined whether there was any interaction between children’s age differences and their motivation to watch COAs of various genres.

Observing COAs of Peking, Yue, and Henan opera, we examined whether there was a significant difference between these genres in terms of viewing motivation–related dimensional performance.

We explored whether there were significant differences in the related motivational performance elicited by children of different ages after viewing different COAs.

## Methods

### Research planning

**Fig 1:**
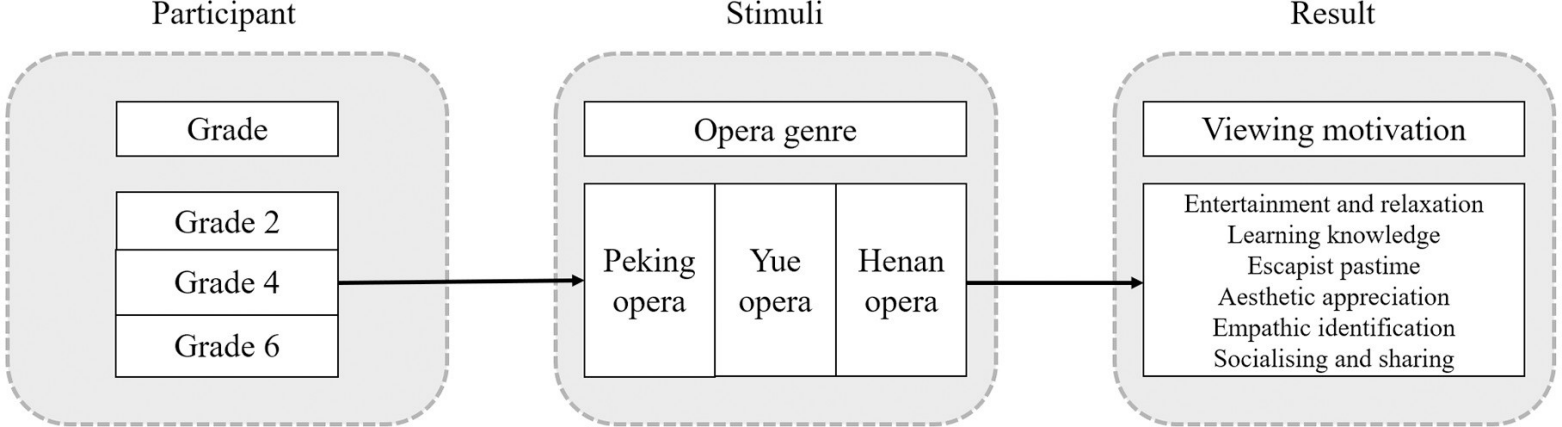
Research structure.

To investigate schoolchildren’s motivation for viewing COA, we used a two-way mixed-design ANOVA, in which the independent variables were based on schoolchildren’s grade and the genre of COAs. The former was divided into three groups: second grade, fourth grade, and sixth grade. The latter comprised three genres: Peking opera, Yue opera, and Henan opera. The dependent variable, “viewing motivation,” included six dimensions: entertainment and relaxation, learning knowledge, escapist pastime, aesthetic appreciation, empathic identification, and socializing and sharing. Fig 1 presents the overall structure of the study. ANOVA was used to determine whether there were differences in the viewing motivation of different schoolchildren when viewing different types of COAs.

### Sampling of participants

Based on existing research [30, 33, 34], we set the age range of child viewers of COA to 6–12 years old, which corresponds to elementary school. The children in this range are in a dynamic growth stage, which can facilitate a better understanding of the motivation of children of different ages to watch COA. Our experiment was conducted at Wuyishan Experimental Primary School in Fujian, China. Prior to the experiment, on October 7–17, 2022, all of the students’ parents signed an informed consent form that explained the purpose, content, use of data, and risks of the experiment. After these forms were obtained, a total of 470 primary school students without a professional background in opera were recruited to participate in the experiment. Finally, 457 valid questionnaires were collected, including 153 students aged 8–9 in second grade, consisting of 87 boys (56.9%) and 66 girls (43.1%); 149 students aged 10–11 in fourth grade, consisting of 83 boys (55.7%) and 66 girls (44.3%); and 155 students aged 11–12 in sixth grade, consisting of 75 boys (48.4%) and 80 girls (51.6%). Participation in the experiment was voluntary and anonymous, and participants could withdraw at any time without reason. No personal information could be identified in the collected data; thus, the questionnaire data were not destroyed after the experiment.

### Selection and testing of stimuli

Based on the seven identified categories of COA themes (i.e., folk life, historical and patriotic, antifeudal oppression, honest and upright official, ethical and moral, love, gods and monsters), we selected the Chinese Opera Classics Original Animation project discussed in the section titled “Chinese opera animation as cultural sustainability.” This series of COAs encompasses an extensive and complete range of opera genres and themes. We first chose animations of 20 minutes or less so that testing could be completed within 40 minutes of classroom time; this is also in line with the typical duration of children’s attention at this stage [37]. The 45 selected animations encompassed the genres of Peking, Yue, and Henan opera. Finally, the story themes were categorized by three experts, consisting of one opera theorist who had been teaching Chinese opera theories for 15 years, one teacher who had been engaged in research on children’s arts for 15 years, and one COA teacher who had been engaged in the creation of COA for 10 years. Based on the experts’ classifications of themes, Table 2 summarizes the distribution of stimuli.

**Table 2:** Classification and integration of stimuli related to Chinese opera genres and story themes.

Theme	Peking opera	Yue opera	Henan opera
Folk life	*Little Cowboy*	*The Jiujin Girl*	*Chao Yang Village*, *Hua Mulan*
Historical and patriotic	*Madam Yue Tattoos Her Son*, *The Legend of the Red Lantern*, *The Taking of Tiger Mountain*, *Xu Ce Running Through Town*, *Mu Guiying Takes Command*, *Sanchakou*, *General and Premier Make Up*, *Empty Fort Strategy*	*Lu Wenlong Returned to the Song Dynasty*	*Sima Yi Explored the Mountains*, *She Taijun Went to War at the Age of 100*
Honest and upright official	*The Case of Chen Shimei*, *Beating the Dragon Robe*, *Honorable Official Yu Chenglong*	*Mermaid Legend*	*Sesame Official*
Love	*The Red Maid*, *Legend of the White Snake*	*Daiyu Enters the Mansion*, *Daiyu Buries Fallen Flowers*, *Daiyu Burned the Manuscript*, *Butterfly Lovers*, *Eighteen Phases Sending*, *Luyou and Tangwan*, *Prince of the Desert*: *Sigh the Moon*, *Biography of Liu Yi*	
Antifeudal oppression	*Lin Chong the Outlaw*, *Su-San Under Police Escort*, *Beat the Stick out of the Box*, *Rescuing the Orphan of Zhao*	*Raccoon for a Prince*, *Meng Lijun*	
Ethical and moral	*Silang Visits His Mother*, *Li Kui Visits His Mother*, *Shi Qian Steals the Chicken*, *Taming of the Princess*, *Escorting Jingniang on a Thousand-Mile Journey*	*He Wenxiu Seeking out His Wife at the Mulberry Orchard*, *Fortune-Telling from He Wenxiu*	
Gods and monsters	*Play Tricks on Pigsy*	*Breaking Open the Mountain to Rescue Mother*	

We can see in Table 2 that the story types of folk life, historical and patriotic, and honest and upright official were present in the COA works in the three key genres. Since the viewers were children, the expert group suggested using folk life stories as a common theme for the stimuli to reduce bias effects caused by differences in story types. Therefore, three works—*Little Cowboy*, *The Jiujin Girl*, and *Hua Mulan*—were chosen as representative COA stimuli for Peking, Yue, and Henan opera. The steps of the experiment are described as follows:

1. Parental informed consent was obtained prior to the experiment. Subjects participated in the experiment on a voluntary basis. The experiment took a class as a unit, and the duration was one class (40 minutes) per week for a total of nine weeks.
2. COA works were played randomly during art class in the classes participating in the experiment, starting on the week immediately after informed consent was obtained. The teachers gave an overview of the work prior to viewing, and the questionnaires were explained and completed after viewing. The researchers and four trained research assistants were responsible for distributing, reading, and collecting the questionnaires.
3. Five children were randomly selected from each participating class according to their student number for semi-structured interviews after the questionnaires were completed. These interviews aimed to check for items given a high score of 4/5 points or a low one of 1/2 points. Subjects were invited to briefly explain the reasons for their scoring to better understand the viewing motivation results.
4. The results of each experiment were recorded on the same day. ANOVA was conducted after the completion of the experiment to see whether there were differences in the performance of viewing motivation in different situations.
5. After the completion of the overall experiment, the reasons for the results were summarized through semi-structured interviews with randomly selected students.

### Questionnaire design

We measured viewing motivation in six dimensions, with five items for each dimension, totaling 30 items (Table 3). We determined the reliability and validity of the questionnaire as follows: (1) the three abovementioned experts were asked to review all questionnaire items to ensure their validity, and (2) a pretest experiment was conducted with 40 children aged 6–12 to check the consistency of the questionnaire through item analysis and reliability testing. Table 3 lists the pretest results. We can see that the Cronbach’s alpha for each of the six dimensions is higher than the required value of 0.7 [106]. After viewing COAs, the subjects scored each item in the questionnaire on a Likert scale. The higher the score, the stronger the viewing motivation.

**Table 3:** Measurement items for each aspect of viewing motivation.

Dimension	Items	Cronbach’s alpha
Entertainment and relaxation	Watching COA can make me feel happy and relaxed	0.914
	I watch COA because I think the content is interesting	
	Watching COA can satisfy my curiosity	
	After watching a COA, I feel like viewing it again	
	Watching COA makes me feel companionable	
Learning knowledge	I can learn about opera from COA	0.923
	I can learn some truths from COA	
	I can learn how to solve the difficulties in life from COA	
	I can learn different life lessons from COA	
	I can learn how to get along with people from COA	
Escapist pastime	Watching COA allows me to temporarily escape from real life, study pressure, and worries	0.897
	I watch COA to pass the time	
	Viewing COA can reduce my loneliness	
	When I have nothing else to do, I like to watch COA	
	When I’m bored, I like to watch COA	
Aesthetic appreciation	I like the cartoon characters in COA	0.932
	The story content in the COA is very attractive to me	
	The images and scenes in the COA appealed to my eyes	
	The music in the COA is good and characteristic, and the dialogue and the overall soundtrack are also very appropriate	
	I like the exaggerated and humorous body movements of the characters in COA	
Empathic identification	I really like the characters in COA; I want to experience their same life	0.915
	Watching COA can satisfy my fantasy	
	I can better understand myself through the cartoon characters in COA	
	Watching COA, I am happy because of the characters’ success and sad because of their failure	
	I admire my favorite characters in COA and can learn to imitate them in my life	
Socializing and sharing	I like to watch COA with my classmates and friends.	0.920
	Watching COA can increase conversations with classmates and friends	
	I like to recommend and share COA to my family and friends	
	Watching COA can help me find friends with common interests	
	I like to talk about COA with people	

### Ethics statement

The study complied with the IRB’s principles and was approved by the Ethics Committee of Jiaxing University. Before data collection, all participants were informed of the benefits, risks, and purpose of the study, as well as how the data would be used. All tests were conducted with the participants’ consent, and the questionnaire was completed anonymously.

## Statistical analysis

Table 4 shows for viewing motivation in each dimension obtained from the experiment. It also lists the differences in different opera genres and in students in different grades. The average of the scores indicates that the viewing motivation induced in children in grades 2 and 4 was higher than that in grade 6. In addition, COA’s effect on inducing motivation for viewing Peking opera was lower than that for viewing Yue and Henan opera. In the following sections, we analyze viewing motivation in each dimension, since two-way ANOVA is needed to determine whether there are significant differences between them.

**Table 4:** Descriptive statistics for the dimensions of viewing motivation.

	Opera	Grade	M	SD	N		Opera	Grade	M	SD	N
ENT	P	2	3.894	.672	153	AES	P	2	3.718	.823	153
		4	3.968	.730	150			4	4.011	.735	150
		6	3.717	.673	154			6	3.636	.746	154
	Y	2	4.077	.480	153		Y	2	3.858	.639	153
		4	4.193	.551	150			4	4.179	.621	150
		6	3.745	.713	154			6	3.623	.797	154
	H	2	3.860	.665	153		H	2	3.765	.744	153
		4	4.152	.544	150			4	4.161	.690	150
		6	3.825	.677	154			6	3.769	.711	154
KNO	P	2	3.754	.729	153	EMP	P	2	3.137	.867	153
		4	3.696	.740	150			4	3.104	.903	150
		6	3.474	.667	154			6	2.764	.791	154
	Y	2	3.852	.642	153		Y	2	3.305	.782	153
		4	3.895	.685	150			4	3.129	.773	150
		6	3.614	.733	154			6	2.800	.857	154
	H	2	3.822	.646	153		H	2	3.252	.794	153
		4	3.863	.690	150			4	3.244	.807	150
		6	3.481	.704	154			6	2.906	.836	154
ESC	P	2	3.603	.676	153	SOC	P	2	3.767	.734	153
		4	3.465	.682	150			4	3.880	.833	150
		6	3.345	.677	154			6	3.651	.800	154
	Y	2	3.749	.645	153		Y	2	3.791	.653	153
		4	3.519	.573	150			4	3.883	.759	150
		6	3.239	.670	154			6	3.552	.890	154
	H	2	3.608	.683	153		H	2	3.859	.687	153
		4	3.552	.641	150			4	4.012	.737	150
		6	3.408	.674	154			6	3.666	.843	154

### ANOVA of entertainment and relaxation (ENT)

Table 5 summarizes the two-way ANOVA of the ENT dimension of viewing motivation. The interaction between the independent variables of COA opera genre and grade level achieved significance (F = 4.415, p = .002). Thus, the effects of the two independent variables on the dependent variable needed to be examined one by one through the simple main effects.

**Table 5:** Summary of two-way ANOVA of the ENT dimension.

Source	Type III SS	df	MS	F	p
Grade	26.729	2	13.364	18.955	.000
Opera	4.898	2	2.449	9.402	.000
Opera * grade	4.600	4	1.150	4.415	.002
	556.619	1362			
Error	32.092	454	.705		
Error (opera)	236.527	908	.260		

**Fig 2:**
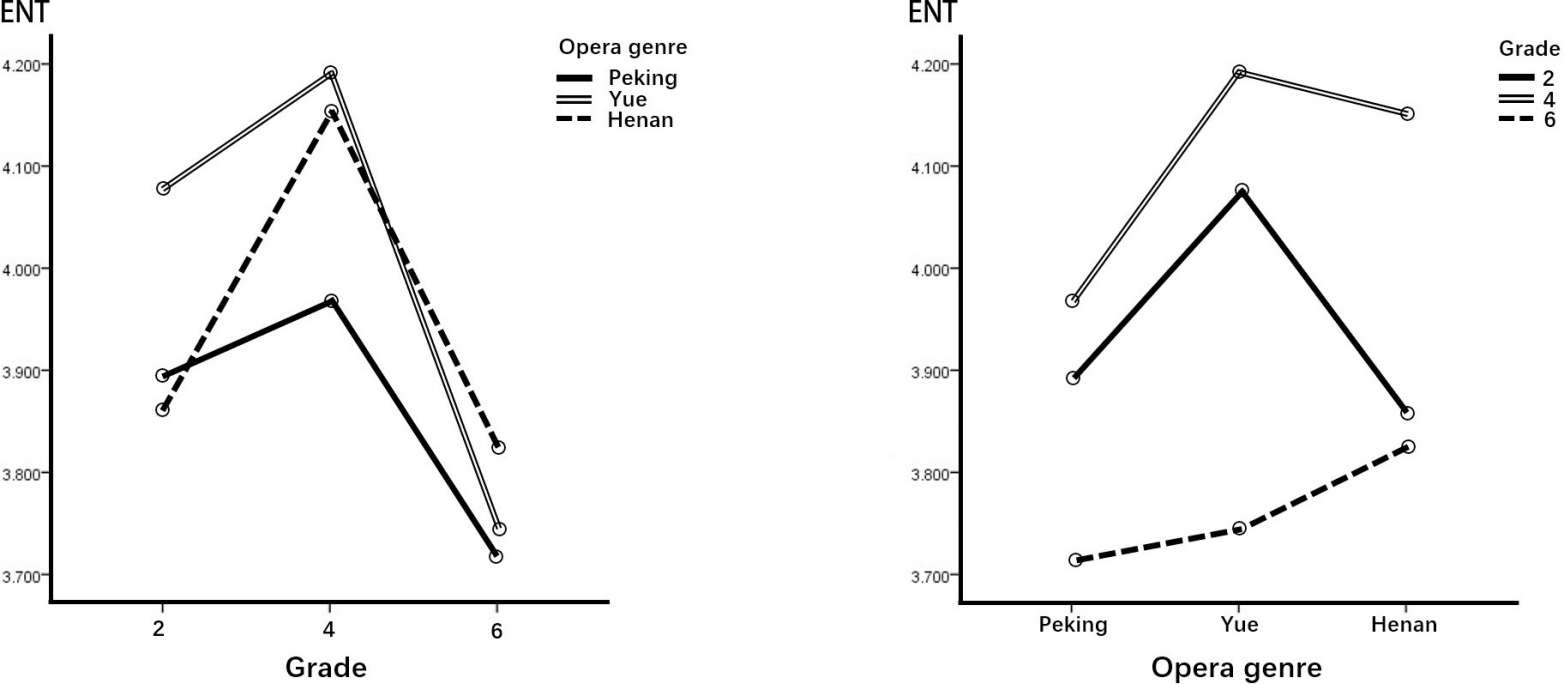
Performance of the ENT dimension.

Table 6 presents the simple main effects based on grade and opera genre. In combination with Fig 2, we can see that except for students in grade 6, the effect of Yue COA was significantly higher than that of Peking COA on students in grades 2 and 4 in the dimension of ENT. The effect of Peking and Yue COAs on children in grades 2 and 4 was significantly greater than that in grade 6 in the dimension of ENT from the perspective of students’ grades. The effect of Henan COA on children in grade 2 was lower than that in grade 4 in the dimension of ENT.

**Table 6:** Simple main effect analysis and post hoc comparisons of the ENT dimension.

Simple main effect		Type III SS	df	MS	F	p	Post hoc
Opera	Grade 2	4.168	2	2.084	8.015	.000	Y > H, P
	Grade 4	4.317	2	2.158	8.300	.000	Y, H > P
	Grade 6	.961	2	.480	1.846	.158	
	Error (residual)	236.527	908	.260			
Grade	Peking	5.535	2	2.767	6.734	.001	4, 2 > 6
	Yue	16.057	2	8.029	19.541	.000	4, 2 > 6
	Henan	9.759	2	4.879	11.875	.000	4 > 2, 6
	Error (residual)	556.619	1362	.411			

### ANOVA of learning knowledge (KNO)

**Fig 3:**
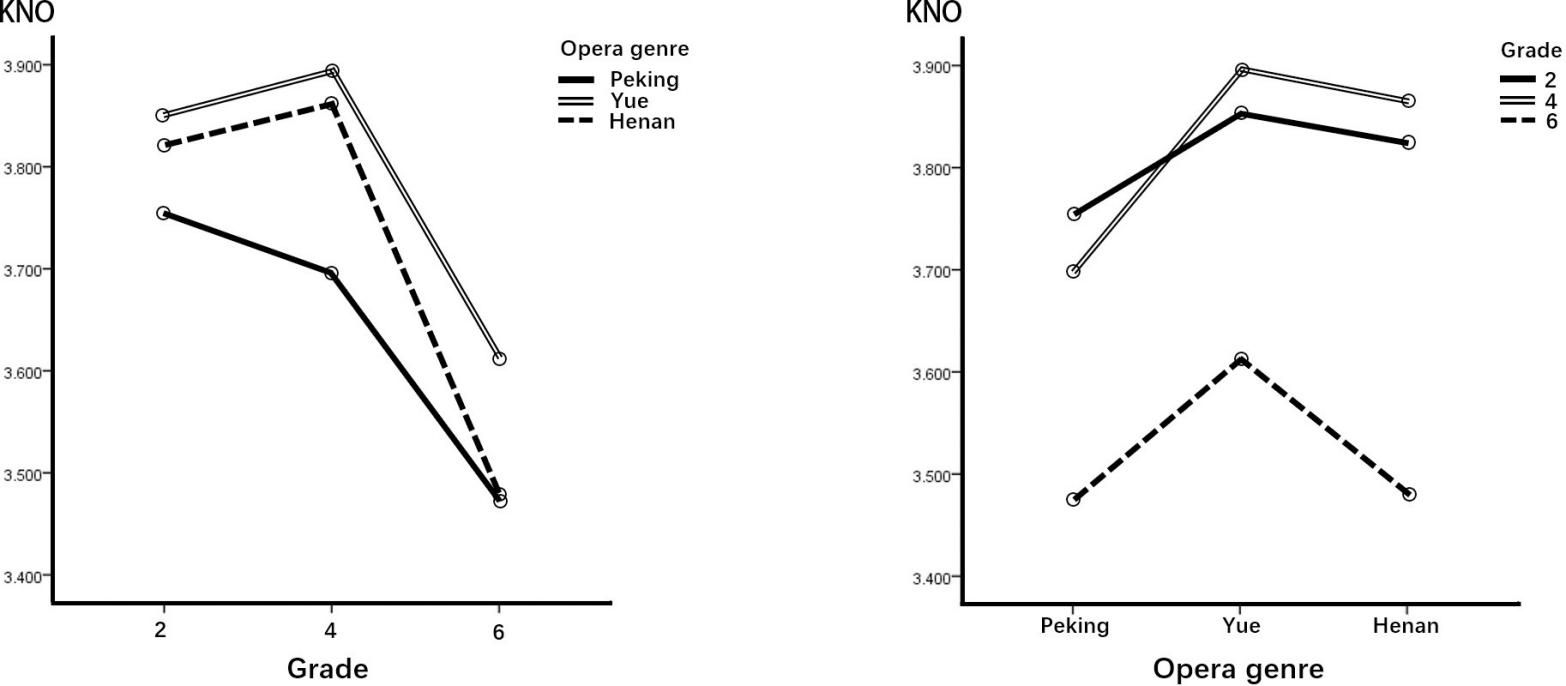
Performance of the KNO dimension.

Table 7 shows the two-way ANOVA of the KNO dimension of viewing motivation. The interaction between the two independent variables of opera genre and grade was insignificant (F = .905, p = .460). This implies that an interpretation can be made directly from the data regarding the main effects of the independent variables. There were significant differences in the effect of COA in the dimension of KNO between opera genre (F = 6.894, p = .001) and grade (F = 17.549, p = .000). This indicates that these two independent variables were influential factors for the dependent variables. The post hoc comparisons in Table 7 and the further inspection in Fig 3 show that the effect of Yue and Henan COAs was higher than that of Peking COA on viewing motivation in the dimension of KNO, and students in grades 2 and 4 showed higher motivation for viewing COA in the dimension of KNO than those in grade 6.

**Table 7:** Summary of two-way ANOVA and post hoc comparisons the KNO dimension.

Source	Type III SS	df	MS	F	p	Post hoc
Grade	25.900	2	12.950	17.549	.000	4, 2 > 6
Opera	4.864	2	2.432	6.894	.001	Y, H > P
Opera*grade	1.277	4	.319	.905	.460	
	655.356	1362				
Error	335.019	454	.738			
Error (opera)	320.337	908	.353			

### ANOVA of escapist pastime (ESC)

Table 8 shows the two-way ANOVA of the ESC dimension of viewing motivation. There was a significant interaction between the two independent variables of opera genre and grade (F = 3.702, p = .005). It was necessary, therefore, to examine the state of differences between the situations through the simple main effect.

**Table 8:** Summary of two-way ANOVA of the ESC dimension.

Source	Type III SS	df	MS	F	p
Grade	24.064	2	12.032	16.750	.000
Opera	.613	2	.306	1.049	.351
Opera * grade	4.322	4	1.081	3.702	.005
	591.193	1362			
Error	326.122	454	.718		
Error (opera)	265.071	908	.292		

**Fig 4:**
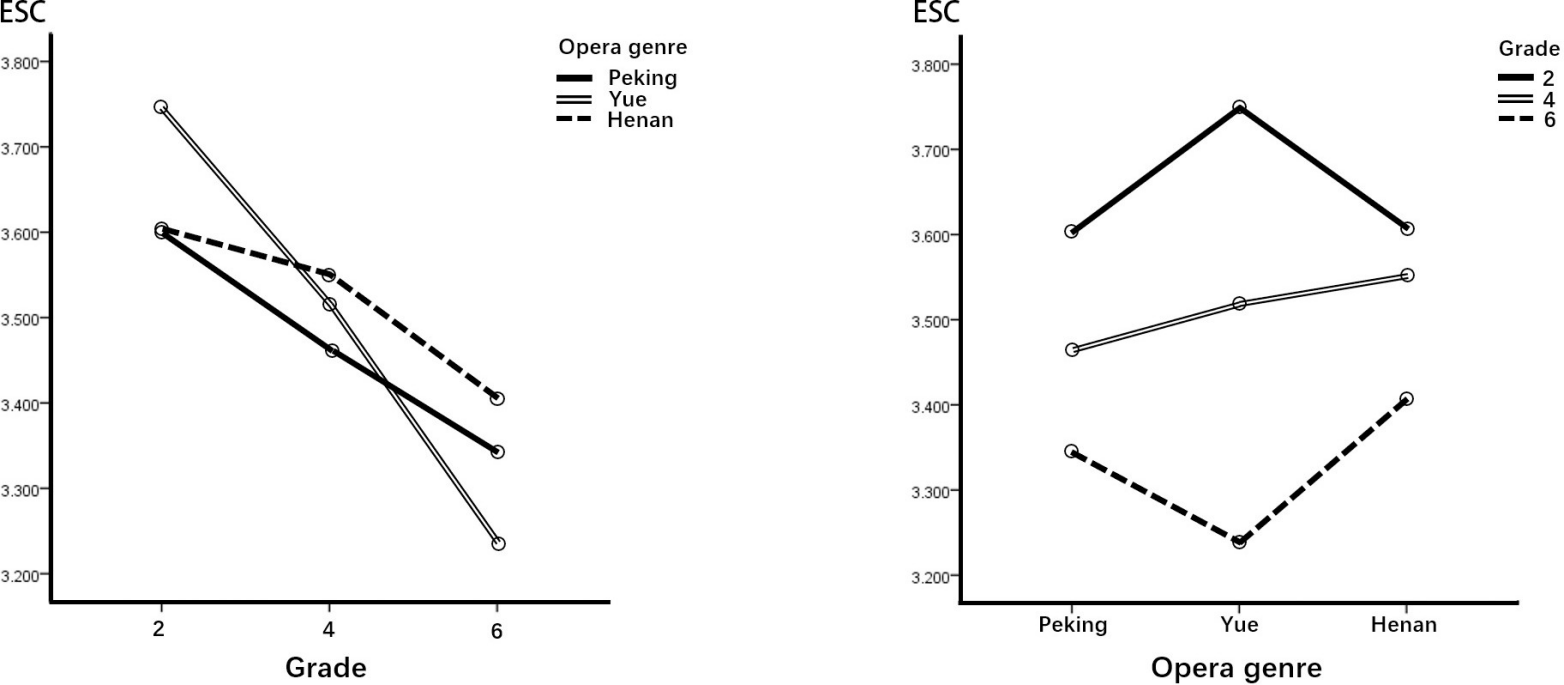
Performance of the ESC dimension.

Regarding grade differences, Table 9 and Fig 4 shows similar results for the three opera genres, with lower grade being significantly higher than the upper grade in terms of ESC, and the middle grade is in between. There were relatively large differences in the performance of opera genre. The lower grade showed significantly higher motivation for ESC in Yue COA than in other genres while the middle grade showed no significant differences among the three genres. The upper grade showed significantly lower motivation toward ESC in Yue opera than in the other two genres.

**Table 9:** Simple main effect analysis and post hoc comparisons of the ESC dimension.

Simple main effect		Type III SS	df	MS	F	p	Post hoc
Opera	Grade 2	2.111	2	1.056	3.616	.027	Y > H, P
	Grade 4	.573	2	.287	.983	.375	
	Grade 6	2.245	2	1.122	3.842	.022	H, P > Y
	Error (residual)	265.071	908	.292			
Grade	Peking	5.391	2	2.696	6.212	.002	2, 4 > 4, 6
	Yue	20.421	2	10.210	23.525	.000	2 > 4 > 6
	Henan	3.271	2	1.636	3.770	.023	2, 4 > 6
	Error (residual)	591.193	1362	.434			

### ANOVA of aesthetic appreciation (AES)

**Fig 5:**
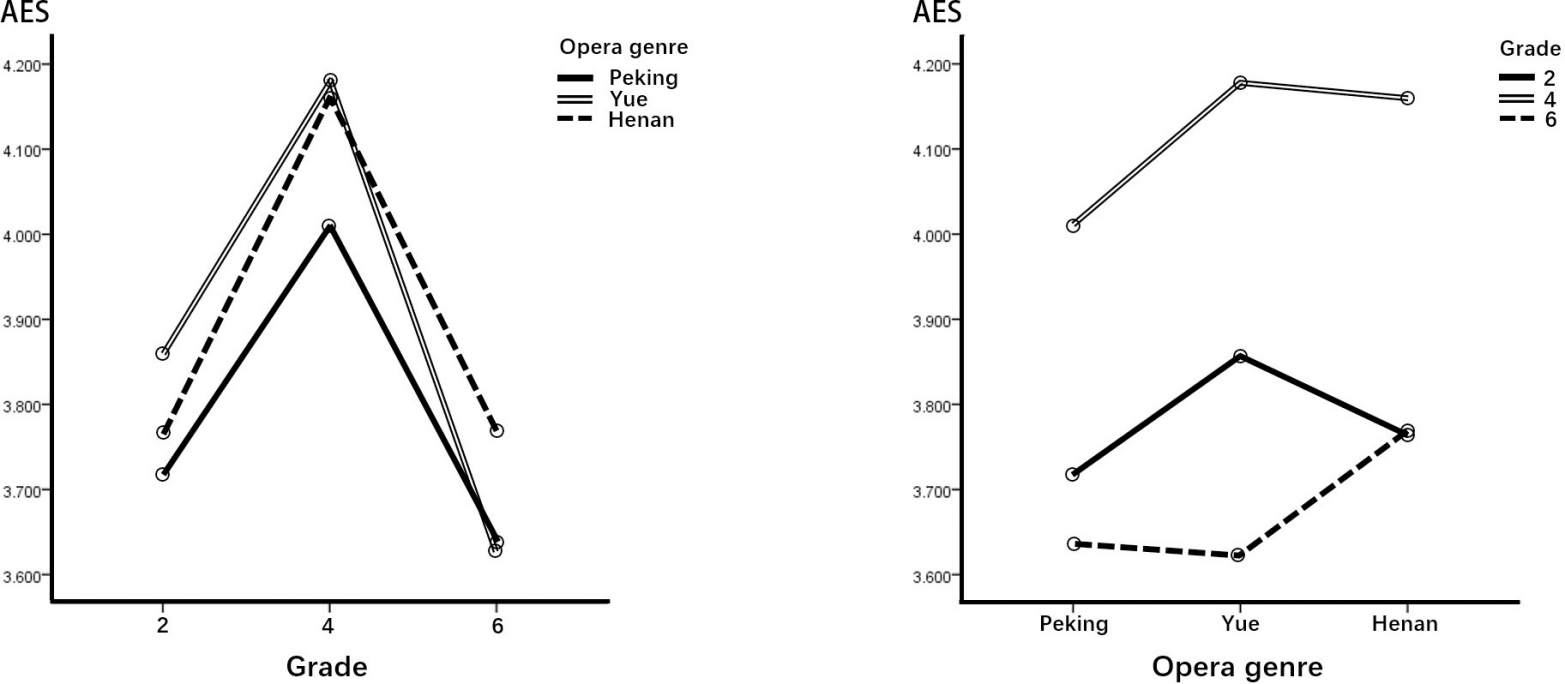
Performance of the AES dimension.

Table 10 shows the two-way ANOVA results based on opera genre and grade in the dimension of AES. We can see that there is no significant interaction between these two independent variables (F = 1.990, p = .094). Therefore, the main effects tested based on both opera genre (F = 4.753, p = .009) and grade (F = 17.549, p = .000) will cause significant differences in this dimension. Fig 5 and the post hoc comparisons in Table 10 show that the Henan and Yue COA stimuli were stronger than Peking COA with regard to viewing motivation in the AES dimension. In addition, students in grade 4 showed a significantly higher motivation for viewing COA in this dimension compared with students in grades 2 and 6.

**Table 10:** Summary of two-way ANOVA and post hoc comparisons of the AES dimension.

Source	Type III SS	df	MS	F	p	Post hoc
Grade	48.219	2	24.110	27.441	.000	4 > 2, 6
Opera	3.338	2	1.669	4.753	.009	H, Y > P
Opera*grade	2.795	4	.699	1.990	.094	
	717.719	1362				
Error	398.892	454	.879			
Error (opera)	318.827	908	.351			

### ANOVA of empathic identification (EMP)

**Fig 6:**
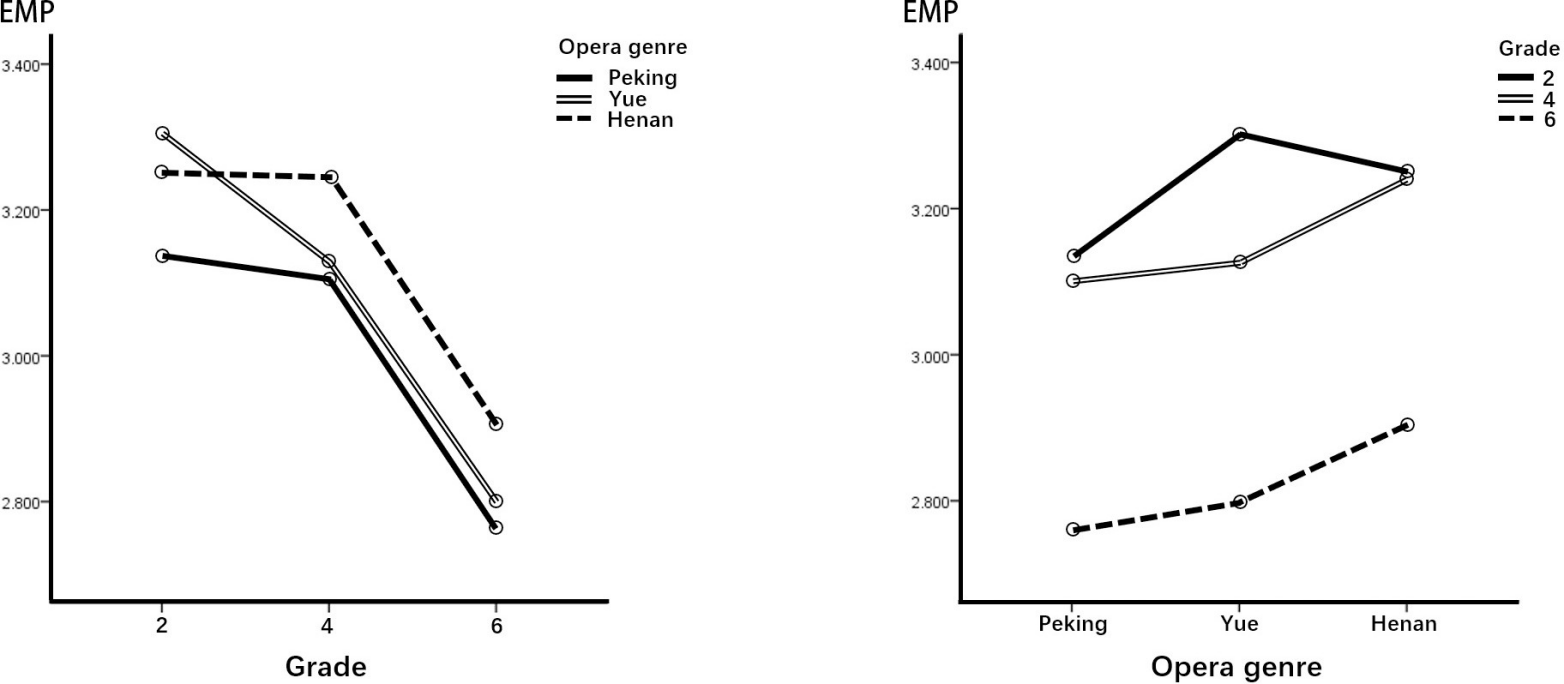
Performance of the EMP dimension.

Table 11 shows the two-way ANOVA results in the dimension of EMP. We can see that there is no significant interaction between the independent variables (F = .878, p = .476). The main effects tested based on both opera genre (F = 4.545, p = .011) and grade (F = 18.984, p = .000) revealed significant effects of COA in the dimension of EMP. Fig 6 and the post hoc comparisons in Table 11 show that Peking COA’s effect on viewing motivation was significantly lower than that of Henan and Yue COAs in the dimension of EMP. Moreover, students in grades 2 and 4 showed a higher motivation for viewing COA in this dimension compared with students in grade 6.

**Table 11:** Summary of two-way ANOVA and post hoc comparisons of the EMP dimension.

Source	Type III SS	df	MS	F	p	Post hoc
Grade	43.638	2	21.819	18.984	.000	2, 4 > 6
Opera	4.040	2	2.020	4.545	.011	H, Y > P
Opera*grade	1.561	4	.390	.878	.476	
	925.378	1362				
Error	521.795	454	1.149			
Error (opera)	403.583	908	.444			

### ANOVA of socializing and sharing (SOC)

**Fig 7:**
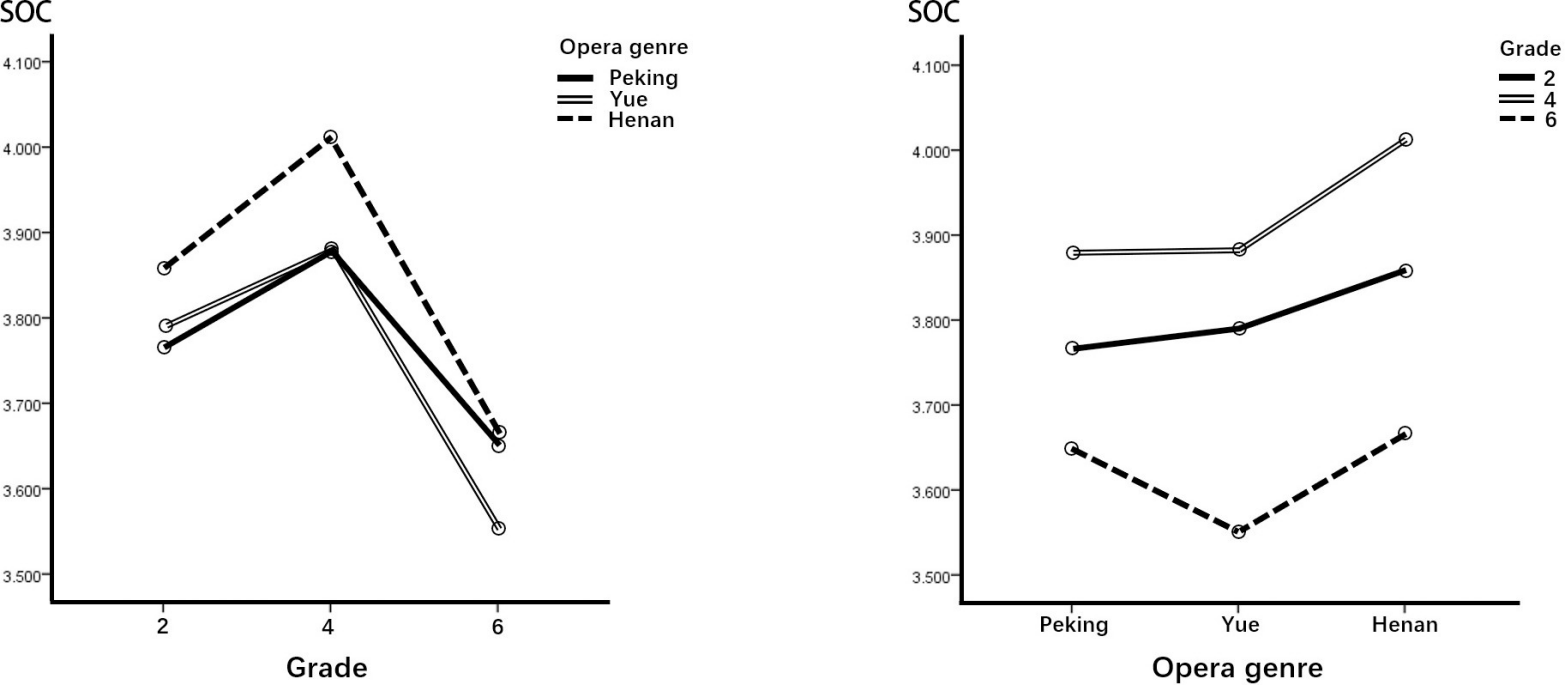
Performance of the SOC dimension.

The two-way ANOVA in Table 12 shows that there is no significant interaction between the independent variables (F = .595, p = .666) in the dimension of SOC. The main effects tested based on both opera genre (F = 3.587, p = .028) and grade (F = 10.108, p = .000) reveal significant differences in the dimension of SOC. Fig 7 and the post hoc comparisons in Table 12 show that the effect of Henan COA was obviously stronger than that of Yue COA on viewing motivation in the dimension of SOC, while the effect of Peking COA was between the two. Students in grade 6 showed obviously lower motivation for viewing COA compared with students in grades 2 and 4.

**Table 12:** Summary of two-way ANOVA and post hoc comparisons of the SOC dimension.

Source	Type III SS	df	MS	F	p	Post hoc
Grade	21.131	2	10.565	10.108	.000	4, 2 > 6
Opera	2.700	2	1.350	3.587	.028	H, P > P, Y
Opera*grade	.896	4	.224	.595	.666	
	816.221	1362				
Error	474.550	454	1.045			
Error (opera)	341.671	908	.376			

### ANOVA of overall viewing motivation

**Fig 8:**
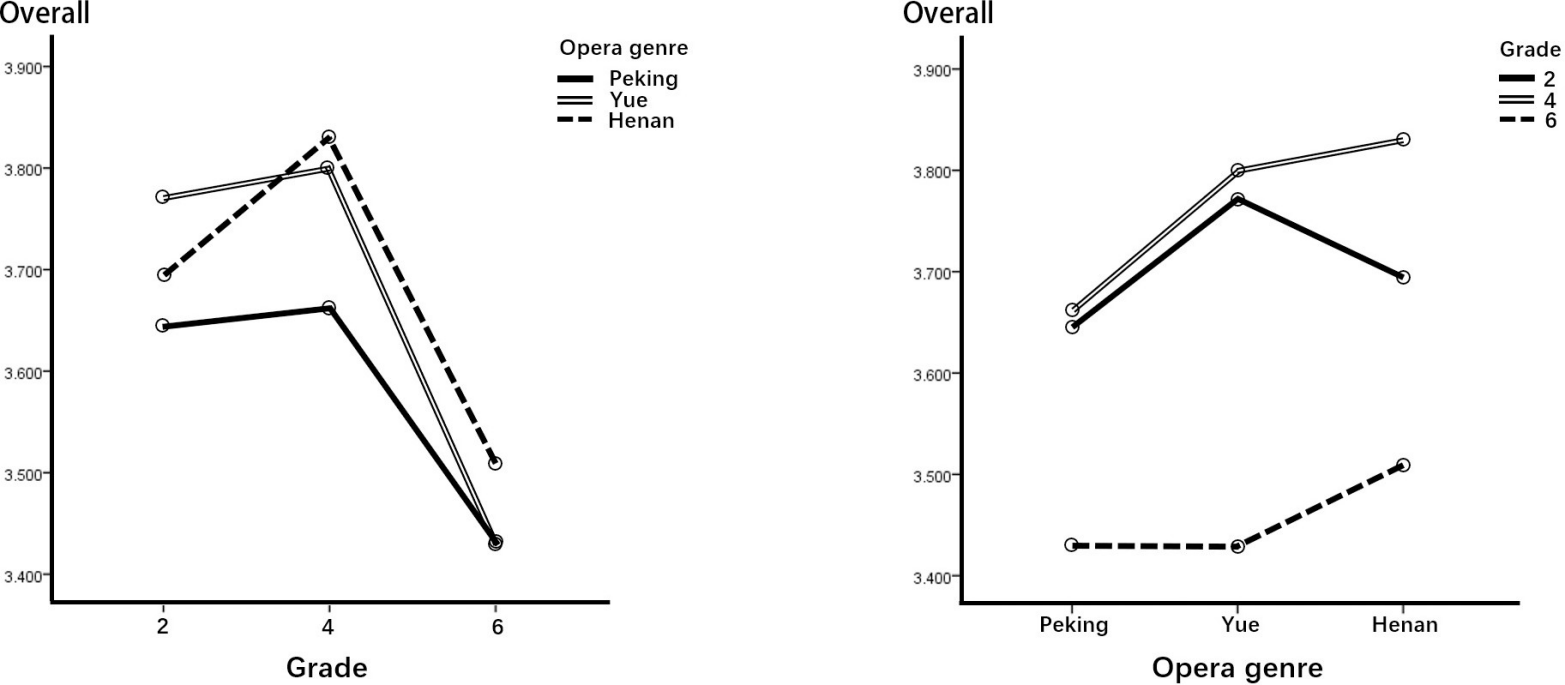
Performance of viewing motivation as a whole.

We examined the overall performance of viewing motive by averaging the data for the relevant dimensions. The two-way ANOVA results in Table 13 show that the interaction did not achieve significance (F = .2.220, p = .065). The main effects tested based on both opera genre (F = 7.043, p = .001) and grade (F = 22.455, p = .000) showed a significant difference in the overall performance of motivation. Fig 8 and the post hoc comparisons in Table 13 show that the students had relatively higher motivation to view Henan and Yue COAs than to view Peking COA. Furthermore, students in grades 2 and 4 showed higher motivation to view COAs than students in grade 6.

**Table 13:** Summary of two-way ANOVA and post hoc comparisons of viewing motivation overall.

Source	Type III SS	df	MS	F	p	Post hoc
Grade	24.407	2	12.204	22.455	.000	2, 4 > 6
Opera	2.638	2	1.319	7.043	.001	H, Y > P
Opera*grade	1.662	4	.416	2.220	.065	
	263.756	1362				
Error	246.740	454	.543			
Error (opera)	17.025	908	.187			

## Discussion and conclusion

### Discussion

Table 14 combines the results of the two-way ANOVAs of COA viewing motives. We can see that there were significant interactions between grade level and COA opera genre in the dimensions of ENT and ESC. The remaining four dimensions of motivation (KNO, AES, EMP, and SOC) did not have significant interactions and can be analyzed from the perspective of grade level and opera genre differences, as discussed below.

**Table 14:** Summary of all comparisons of COA viewing motivation.

	ENT	KNO	ESC	AES	EMP	SOC	Overall
Grade		4, 2 > 6		4 > 2, 6	2, 4 > 6	4, 2 > 6	4, 2 > 6
Peking	4, 2 > 6		2, 4 > 4, 6				
Yue	4, 2 > 6		2 > 4 > 6				
Henan	4 > 2, 6		2, 4 > 6				
Opera		Y, H > P		H, Y > P	H, Y > P	H, P > P, Y	Y, H > P
Grade 2	Y > P, H		Y > H, P				
Grade 4	Y, H > P		ns				
Grade 6	ns		H, P > Y				

First, regarding ENT, children in grades 2 and 4 (lower and middle) were significantly more likely to be entertained and relaxed by Yue COA than by Peking COA. In the posttest interviews, most of the lower- and middle-grade schoolchildren found the story interesting, liked the characters’ personalities, and found the combination of opera singing and story appropriate. Schoolchildren in grade 6 (upper) had the lowest scores among the three grades regarding motivation to view COA, but still showed positive attitudes in the dimension of ENT. The middle-grade group experienced the most entertaining and relaxing effects when watching COAs. Yue and Peking COAs are suggested as a good option for inducing motivation in this area for lower-grade students. Second, regarding the ESC dimension, children in grade 2 felt significantly more positive about this dimension than the older children in grade 6. It is noteworthy that Yue COA offered the best escapism for younger children but was the least evocative of all opera genres for the older children. In posttest interviews, some sixth graders said they found the stories and plots simple and tedious and would therefore choose other ways to pass the time.

Regarding the three dimensions of KNO, EMP, SOC, these motives were induced more strongly in lower- and middle-grade groups than in the upper-grade group. In the interviews, children in grades 2 and 4 said that, because they liked the story content and characterization, they could learn good qualities, like those of the main character, and also acquire some opera knowledge and life experience. After watching COAs, their acceptance of traditional opera improved, and they were willing to recommend COA to their families and friends. By contrast, the upper-grade children found the COA stories less intellectual and story content; thus, they were less willing to share. In terms of AES motivation, lower- and upper-grade children were less motivated than the middle-grade group. During the interviews, the fourth-graders (middle grade) thought the characters in the COA looked good, the music had character, and the singing was fun. Second graders paid more attention to story and characterization and had no particular view regarding AES. Sixth graders emphasized the need for more detail in the shape and movement of animated characters and in the design of scenes. Therefore, in terms of the overall performance of viewing motivation, the second and fourth grades can be considered relatively suitable stages for introducing COA to schoolchildren.

With regard to opera genre differences, in terms of KNO, EMP, and AES, Yue and Henan COAs had better motivational effects than Peking opera. In terms of SOC, however, Yue opera was the least effective among the three genres. Through the interviews, we learned that some children liked the stories of the Yue and HenanCOAs as well as the proper integration of characters with their personalities and singing voices. They felt they could empathize with the characters and also thought the animations were too short, prompting them to ask further questions about them. Meanwhile, regarding the Peking opera COA, they suggested improving the story, and they felt there was too much singing and not enough story content and action. Thus, our findings indicated that, compared with Peking opera, animations of Yue and Henan opera are more likely to stimulate children’s motivation for further KNO, EMP, and AES. Animations of Yue and Henan operas can therefore be considered a better choice for enhancing learning knowledge motivation.

It is worth noting that Yue COA had the strongest effect on children’s viewing motivation. Yue opera is a genre mainly involving singing and “gentle shows.” The singing aspect of the opera focuses on lyricism, and the rhythm is relatively slow paced [24]. Large segments of singing in a Chinese opera could make it difficult to understand for children, and they might not readily accept it [24, 36]. Our results suggest otherwise. The interviews indicated that most students did not dislike singing in COAs because they found the stories and characters attractive, and the singing and plots were coordinated in a cohesive way. Specifically, most children in the lower grade indicated that they liked the singing parts in COAs. Those in the middle grade said that longer singing segments would not affect their viewing if the stories were good. Lastly, most children in the high grade said that the singing parts needed to be better integrated with the stories; they also had higher requirements for the design of the story and the characters. This echoes the idea that children entering the formal operational stage from the concrete operational stage are beginning to think for themselves and give more consideration to abstract concepts and propositions [107]. As their attention spans and perceptive capabilities increase, these children will pay more attention to the design details of animations [108]. Thus, the attractiveness of COA stories and the coordination of singing and story will affect children’s acceptance of the singing segments in COAs.

Taken together, among the various parts of a COA, such as story, singing, and acting, the richness and interestingness of the story is what best induces schoolchildren’s motivation to watch COAs. COAs therefore need to be developed according to developmental status of children at different ages. We recommend that for audiences in the fourth grade and beyond, COAs should enhance the dramatic conflict and richness of the story. The content should not be overly simple or childish. In addition, opera knowledge and the artistic features of opera art itself, such as suppositionality, should be integrated appropriately and gradually to enhance the depth of the narrative and the aesthetics of COA for children in the middle and upper grades to avoid making them feel childish and bored. Today’s schoolchildren exist in a different environment from that of opera audiences in the heyday of Chinese opera. Children who do not acquire systematic knowledge about Chinese opera at an early age will lack the basic knowledge needed to appreciate it [21, 28, 29, 36]. It is necessary, therefore, to begin opera education activities in elementary school to promote aesthetics and pass on Chinese opera culture. In terms of teaching, it is advisable to start showing and teaching COAs during the lower and middle grades, and to select materials suitable for the children’s developmental stages so that they can accept and understand the opera genres and themes they like and gradually cultivate a love for Chinese opera. By the time they are seniors, they will have cultivated the habit of watching opera and accumulated aesthetic literacy in opera culture, thus promoting the perpetuation of opera art and culture.

### Conclusion and recommendations

Watching COAs is a type of art-reception activity that can only survive and develop if it is understood and accepted by audiences. Since children are the main audience for COA, it is important to understand their motivation for watching it to achieve the ultimate goal of promoting the preservation of traditional opera culture. This study fills a gap in the research regarding schoolchildren’s motivation to watch COAs. The main findings are summarized as follows:

1. In terms of the motivation for watching COA, Peking and Yue COAs were found to have better entertainment and relaxation benefits for lower- and middle-grade children. Henan COA, meanwhile, had better escapist effects for upper-grade children but had relatively lower effects for lower-grade schoolchildren.
2. Both Yue opera and Henan opera had better motivational triggers than Peking opera in terms of KNO, EMP, and AES. In terms of overall performance, Yue and Henan opera are better motivational choices for COA appreciation.
3. The motivational performance of the middle- and lower-grade groups in terms of KNO, EMP, and SOC was higher than that of the upper-grade group in COA appreciation. Thus, the second and fourth grades are the most appropriate stages for introducing COA appreciation to schoolchildren.

We found that Peking opera was less motivating for children than Yue opera, which is mainly sung, and Henan opera, which has local characteristics. Each opera genre has its own special subject matter. For example, Peking opera has many historical and patriotic plays, with both “gentle shows” and acrobatic military plays. Henan opera has many historical plays, with local characteristics that are simple and popular. Yue opera has mainly literary plays that are mostly sung and focus on family ethics, love, and small folk stories. Therefore, follow-up research could focus on story themes, which could further enrich the study of COA in terms of children’s motivation to watch it.

## Supporting information

S1 File Data for statistical analysis. (XLSX)

S2 File Informed consent of the child’s parent or legal representative. (DOCX)
