# Supplementary material for: Schoolchildren’s motivation for viewing Chinese opera animation according to opera genre
Source: PLoS One. 2023 Oct 11;18(10):e0292744. doi: 10.1371/journal.pone.0292744 (PMC10566701; doi:10.1371/journal.pone.0292744)
Supplement: S2 File — (DOCX) [file pone.0292744.s002.docx]

**Informed consent of the child’s parent/legal representative**

Dear parents/legal representative:

We are a research team from the Department of Design at Chung Yuan University. The research project facilitator is Ms. Lv. We cordially invite your children to an appreciation study of opera animation to help them learn the culture, specifically, the art of Chinese opera. The said study will provide us with the added benefits of being able to understand the viewing motivation and preference of children’s audience for current Chinese opera animation and of acquiring feedback from young viewers. Your support and participation can provide animation creators with more ideas and insights for improvements concerning the production of children’s Chinese opera as well as better aid in the inheritance and promotion of culture in China.

Program: Schoolchildren’s Motivation for Viewing Chinese Opera Animation According to Opera Genre

**Program host affiliation:**

Lv, Department of Design, Chung Yuan University

E-mail: [277540748@qq.com](mailto:277540748@qq.com)

**Our research content:**

Chinese opera is a form of culture with a long history in China but is gradually being forgotten by the younger generation as it loses the audience in large numbers with the rise of the Digital Age. To preserve and promote the culture of Chinese opera, Chinese opera animation was created. Studying children’s viewing motivations of Chinese opera animation can help improve the production and teaching of opera animation.

**Study conducted:**

We are working with the Wuyishan Experimental Primary School to prepare a program on opera animation viewing. The program will be developed in tandem with several integrated arts classes, and the children will watch, learn, and talk about Chinese opera animation during their weekly art classes. At the end of the course, the children will submit a questionnaire about the children’s motivation to watch.

**Your and your child’s identifying information will be kept confidential!**

1. Our research lessons are taught by project facilitators and school teachers working together on the basis of the nature and content of the curriculum. Whether you agree to the participation of your child, your child’s normal learning and academic performance will not be affected.
2. We take an “anonymous” approach, where we replace the child’s real name with a code. We are also responsible for maintaining the confidentiality and will not disclose any information about the children to anyone.

**We will safeguard your child and respect any decision to pull out your child:**

Participation in this study will not cause physical or psychological harm to your child. If you and your child wish to withdraw at any time during the study, we will gladly abide by your decision.

**We will provide a small token gift:**

After the completion of the interview, we will offer small prizes based on the children’s positive performance. In case your child drop out of the study halfway through, we will also provide a small gift as a token of appreciation for sharing your valuable experience.

**How we will use the information your child provides:**

The information provided by the child will be stored on a hard drive or computer with a password, to be deleted and destroyed at the end of the study, and will solely be used for the avowed purposes of this research undertaking. The child’s real name and personal information will not appear on the report when the research results are presented in the future.

**If you have any questions about the above, please feel free to ask. If you agree, please sign below; if you do not agree, there is absolutely no reason to be embarrassed.**

**Parent/legal representative signature field:**

Signature:………………………………………………………………Date:………

**Research Team signature column:**

Signature of the Program Facilitator:…………………………………Date:………

**Two copies of this consent form will be retained by each party for future contact.**

**儿童家长/法定代理人知情同意书**

**亲爱的家长您好：**

我们是台湾中原大学设计学系所的研究团队，研究计划主持人为吕婵老师（武夷学院艺术学院）。我们诚挚地邀请您的孩子进行戏曲动画的赏析学习，帮助孩子们学习传统文化——戏曲艺术，同时协助我们了解儿童观众对于当前戏曲动画的观赏动机、喜好及小观众们的反馈意见。您的支持与参与能够为戏曲与动画创作者们提供更多的儿童戏曲动画创作思路与改善意见，更好的帮助我国传统文化的传承与弘扬。

**计划名称：儿童观众戏曲动画观赏动机研究**

**计划主持人、所属单位：**

台湾中原大学设计学系所 吕婵

1. mail：[277540748@qq.com](mailto:277540748@qq.com)

**我们的研究内容：**

戏曲艺术是我国传统文化，随着数字时代的发展，逐渐被年轻一代遗忘，出现“弦断有谁听”的尴尬境地。将戏曲艺术与孩子们喜欢的动画进行结合，为保护与弘扬中国传统文化、振兴中国民族动画的发展，研究儿童观众戏曲动画，迫切需要了解儿童观众视角下的观赏动机、喜好、想法等相关信息。

**研究进行：**

我们与武夷山市实验小学进行合作，开展传统文化艺术进校园课程。该课程串联美术、音乐等多门综合艺术课程于周三下午在实验小学课堂进行。届时孩子们进行戏曲动画知识学习与鉴赏活动，在课程结束时，提交一份儿童观众观赏动机问卷及简短的访谈。

**您及孩子的资料将受到妥善保密！**

1、我们的研究会由计划主持人与学校老师针对课堂进行研究展开课程，无论您是否同意孩子参加该研究，皆不影响孩子的学习成绩、老师观感。

2、我们采取“匿名”的方式，以编码来取代孩子的真实姓名。我们也会负起责保密责任，不会向任何人透漏有关孩子的资料。

**我们会善尽保护和尊重的责任：**

参与这个研究不会对孩子身体或心理造成伤害，过程中，若想要退出研究，我们会尊重孩子及您的决定。

**致赠小礼物：**

完成学习与访谈后，我们会根据孩子的学习表现提供小奖品；若中途退出访谈，我们也会提供一份小礼物，感谢宝贵的经验分享。

**我们将如何使用您孩子提供的资料：**

孩子所提供的问卷学习资料，我们会妥善保存在设有密码的硬盘或计算机里，且于研究结束删除销毁，并只使用在本研究。未来研究成果呈现时，孩子的真实姓名及个人资料将不会出现在报告上。

**上述内容，您有任何问题，请尽管提问。如同意，请于下方签署；如不同意，也请不用为难！**

**家长/法定代理人签署栏：**

签名： 日期： 年 月 日

**研究团队签署栏：**

计划主持人签名： 日期： 年 月 日
